# Interconnected feedback loops among ESRP1, HAS2, and CD44 regulate epithelial-mesenchymal plasticity in cancer

**DOI:** 10.1063/1.5024874

**Published:** 2018-08-22

**Authors:** Mohit Kumar Jolly, Bogdan-Tiberius Preca, Satyendra C. Tripathi, Dongya Jia, Jason T. George, Samir M. Hanash, Thomas Brabletz, Marc P. Stemmler, Jochen Maurer, Herbert Levine

**Affiliations:** 1 Center for Theoretical Biological Physics, Rice University, Houston, Texas 77030, USA; 2 Department of Experimental Medicine I, Nikolaus-Fiebiger Center for Molecular Medicine, Friedrich-Alexander University of Erlangen-Nürnberg, Erlangen 91054, Germany; 3 Department of General and Visceral Surgery, Medical Center, University of Freiburg, Faculty of Medicine, Freiburg 79106, Germany; 4 Department of Clinical Cancer Prevention, UT MD Anderson Cancer Center, Houston, Texas 77030, USA; 5 Program in Systems, Synthetic, and Physical Biology, Rice University, Houston, Texas 77030, USA; 6 Department of Bioengineering, Rice University, Houston, Texas 77030, USA; 7 Medical Science Training Program, Baylor College of Medicine, Houston, Texas 77030, USA; 8 German Cancer Consortium (DKTK), Heidelberg 69120, Germany; 9 German Cancer Research Center (DKFZ), Heidelberg 69120, Germany

## Abstract

Aberrant activation of epithelial-mesenchymal transition (EMT) in carcinoma cells contributes to increased migration and invasion, metastasis, drug resistance, and tumor-initiating capacity. EMT is not always a binary process; rather, cells may exhibit a hybrid epithelial/mesenchymal (E/M) phenotype. ZEB1—a key transcription factor driving EMT—can both induce and maintain a mesenchymal phenotype. Recent studies have identified two novel autocrine feedback loops utilizing epithelial splicing regulatory protein 1 (ESRP1), hyaluronic acid synthase 2 (HAS2), and CD44 which maintain high levels of ZEB1. However, how the crosstalk between these feedback loops alters the dynamics of epithelial-hybrid-mesenchymal transition remains elusive. Here, using an integrated theoretical-experimental framework, we identify that these feedback loops can enable cells to stably maintain a hybrid E/M phenotype. Moreover, computational analysis identifies the regulation of ESRP1 as a crucial node, a prediction that is validated by experiments showing that knockdown of ESRP1 in stable hybrid E/M H1975 cells drives EMT. Finally, in multiple breast cancer datasets, high levels of ESRP1, ESRP1/HAS2, and ESRP1/ZEB1 correlate with poor prognosis, supporting the relevance of ZEB1/ESRP1 and ZEB1/HAS2 axes in tumor progression. Together, our results unravel how these interconnected feedback loops act in concert to regulate ZEB1 levels and to drive the dynamics of epithelial-hybrid-mesenchymal transition.

## INTRODUCTION

Recent studies have elucidated the significance of dynamic reciprocal relationships among tumor cells and the extracellular matrix (ECM).^1^ The complex nonlinear behavior emerging from interconnected mechanical and chemical multi-scale regulatory feedback loops can modulate many parameters of aggressive cancer behavior such as metastasis, drug resistance, and tumor relapse.^1–3^ Epithelial-mesenchymal transition (EMT) is a latent embryonic program that may become aberrantly activated during tumor progression and can regulate metastasis,^4^ drug resistance,^5^ evasion of the immune system,^6^ and tumor-initiation and relapse.^7,8^ EMT is characterized by its effects on one or more of these traits—decreased cell-cell adhesion, rearranged cytoskeleton, disrupted apico-basal polarity, and increased migration and invasion.^9^ EMT can be induced by multiple micro-environmental conditions such as ECM stiffness and hypoxia^10–13^ which can activate one or more of EMT-driving transcription factors (EMT-TFs) such as ZEB (ZEB1/2), Snail (SNAI1/2), and Twist.^14^ In turn, EMT-TFs such as ZEB1 can crosslink collagen in the ECM, altering mechanical stiffness of the ECM.^15^ Thus, EMT acts as a hub in mediating the mechanochemical response of the tumor microenvironment (TME).

Here, we focus on ZEB1—a key driver of EMT—which can confer tumor-initiating (stemness) and chemoresistance properties to cancer cells,^16,17^ and its expression correlates with poor prognosis.^18^ ZEB1 mainly acts as a transcriptional repressor for genes involved in cell-cell adhesion and cell polarity^14^ and for microRNAs that promote an epithelial phenotype such as the miR-200 family.^19,20^ The miR-200 family also represses ZEB1 translation, thereby forming a mutually inhibitory feedback loop,^21,22^ referred to as the “motor of cellular plasticity.”^23^ The miR-200 family can also directly target multiple ECM proteins,^24^ hence affecting TME mechanics.

**FIG. 1.**
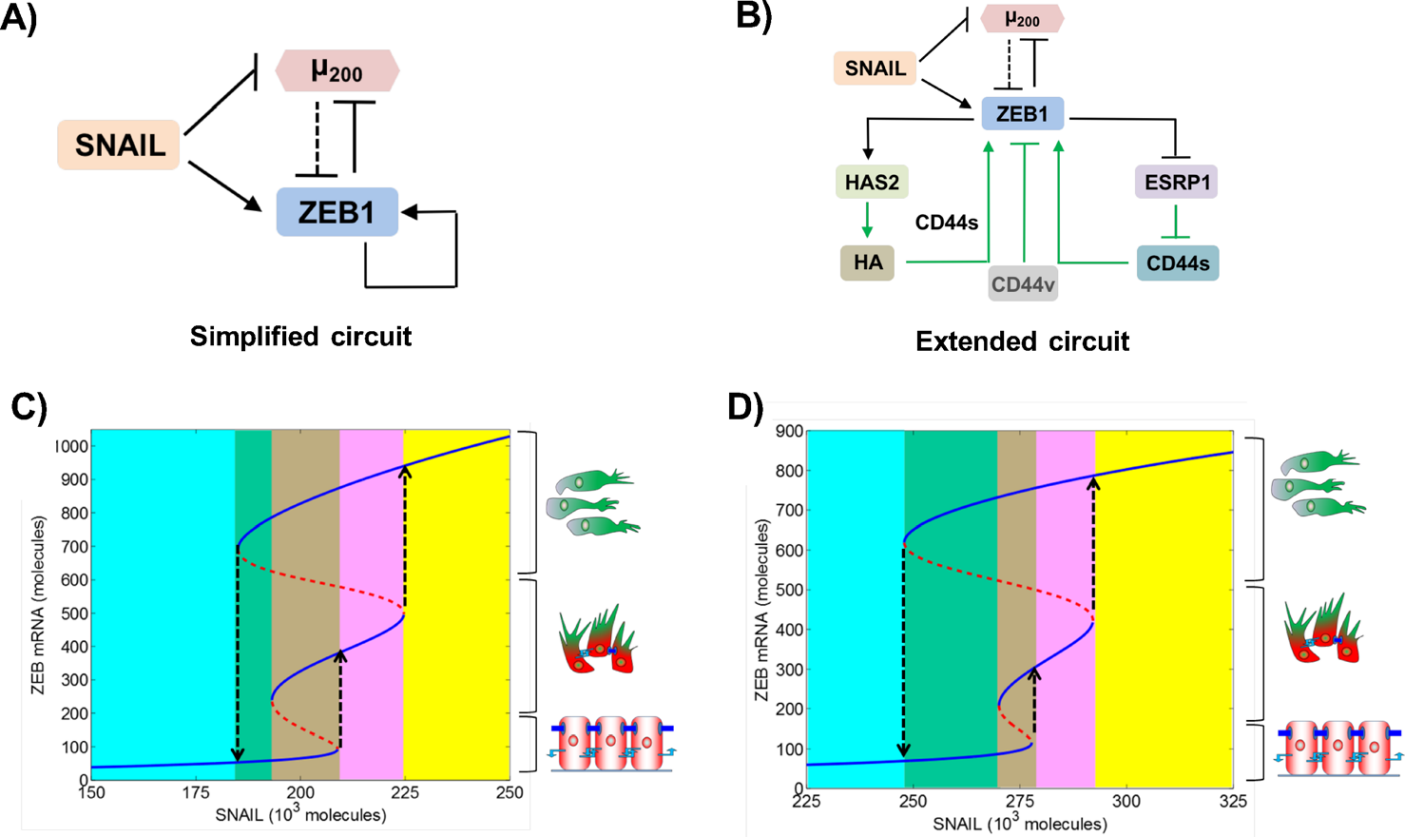
Bifurcation diagrams of simplified and extended EMT regulatory circuits. (a) Simplified EMT regulatory circuit—mutually inhibitory feedback loop between ZEB1 and miR-200, driven by SNAIL. In this earlier modeling attempt, self-activation of ZEB1 is considered to be direct, to incorporate the stabilization of SMADs by ZEB1. (b) Extended EMT regulatory circuit with explicit mechanistic details of ZEB1 self-activation, as identified in recent studies. Solid black lines show transcriptional regulation, dotted black lines show microRNA-mediated regulation, and green arrows indicate regulation whose modes are either non-transcriptional or yet have to be identified. (c) Bifurcation diagram of the miR-200/ZEB1 (simplified) circuit shown in (a), driven by SNAIL. Solid blue curves show stable states (cellular phenotypes), and red dotted curves show unstable states. Low levels of ZEB1 denote an epithelial (E) phenotype, high levels of ZEB1 denote a mesenchymal (M) phenotype, and intermediate levels of ZEB1 denote a hybrid E/M phenotype, as shown by cartoons drawn alongside. Different colored regions show different phases—the cyan region depicts the {E} phase, the green region depicts the {E, M} phase, the brown region depicts the {E, E/M, M} phase, the pink region depicts the {E/M, M} phase, and the yellow region depicts the {M} phase. (d) Bifurcation diagram for the extended, i.e., miR-200/ZEB1/ESRP1/HAS2/CD44, circuit as shown in (b).

Recent *in vitro*, *in vivo*, and patient data have revealed that EMT is rarely an “all-or-none” process; instead, cells can stably display one or more hybrid epithelial/mesenchymal (E/M) phenotype(s).^25–31^ Computational modeling efforts have suggested that the dynamics of the miR-200/ZEB1 loop can allow cells to attain not only an epithelial (high miR-200 and low ZEB) and a mesenchymal (low miR-200 and high ZEB) phenotype^32,33^ but also a hybrid E/M state—(medium miR-200 and medium ZEB).^34–36^ Indeed, active nuclear ZEB1 was observed in H1975 lung cancer cells^37^ that maintain a stable hybrid E/M state at a single-cell level over many passages.^25^ Furthermore, ZEB1 was expressed in pancreatic cancer cells stably maintaining a hybrid E/M state for six months.^30^ These earlier models presumed a self-activation of ZEB1,^34–36^ based on ZEB1-mediated stabilization of SMAD complexes^34^ [Fig. 1(a)]. More recently, exact molecular mechanisms mediating a ZEB1 self-activation have been elucidated.^38,39^

Particularly, ZEB1 can self-activate through two interconnected feedback loops. First, ZEB1 represses epithelial splicing regulatory protein 1 (ESRP1)^39^ that controls alternative splicing of the cell surface receptor CD44 which is differentially regulated during EMT.^40^ Decreased ESRP1 leads to enhanced levels of the CD44s (CD44 standard; devoid of all variable exons) isoform and depleted levels of variable-exon containing CD44v (CD44 variant) isoforms. Overexpression of CD44s, in turn, activates ZEB1, whereas an increase in CD44v isoforms inhibits ZEB1^39^ [Fig. 1(b)]. Second, ZEB1 activates hyaluronic acid synthase 2 (HAS2) that synthesizes hyaluronic acid (HA)—a key proteoglycan in the ECM that, in turn, elevates the levels of ZEB1^38^ [Fig. 1(b)]. These two feedback loops are connected due to interactions of HA with its receptor CD44. CD44s was reported to be essential for the effect of HA on ZEB1, suggesting that HA binding to its receptor CD44s affects EMT.^38^ CD44s-HA interactions have been proposed to protect cells undergoing EMT against anoikis,^41^ a trait commonly associated with EMT,^42^ thereby reinforcing the role of CD44s-HA interactions in this process. CD44s overexpression together with excess of HA leads to even higher levels of ZEB1 as compared to CD44s overexpression alone.^38^ Hence, CD44s may be able to activate ZEB1 through both HA-dependent and HA-independent pathways. However, how these complex interconnections among these feedback loops regulating ZEB1 drive the emergent dynamics of epithelial-hybrid-mesenchymal transitions remains elusive.

Here, using an integrated theoretical-experimental approach, we elucidated the dynamics of the interconnected feedback loops among ZEB1, ESRP1, HAS2, and CD44 in mediating epithelial-hybrid-mesenchymal transitions. First, we constructed a mathematical model denoting the known interactions among these players. The model predicted that these feedback loops can enable the existence of a stable hybrid E/M phenotype, besides epithelial and mesenchymal phenotypes. Sensitivity analyses on model parameters identify ESRP1 as a key mediator of EMT and its reverse mesenchymal-epithelial transition (MET). Moreover, ESRP1 negatively correlated with both ZEB1 and the extent of EMT across multiple datasets corresponding to *in vitro* experiments and primary tumor samples. Consistently, knockdown of ESRP1 in stable hybrid E/M cells—H1975—drove the cells towards a full EMT. Finally, higher levels of ESRP1, ESRP1/HAS2, and ESRP1/ZEB1 correlate with poor patient survival, indicating the functional relevance of these feedback loops during tumor progression.

## RESULTS

### Mathematical modeling suggests how the miR-200/ZEB1/ESRP1/HAS2/CD44 circuit drives epithelial-hybrid-mesenchymal transition

As a first step towards elucidating the characteristics of epithelial-hybrid-mesenchymal transition as driven by ZEB1/ESRP1/HAS2/CD44 feedback loops, we construct a mathematical model representing the experimentally known regulatory interactions among these players (Sec. SI in the supplementary material). Next, we plot the levels of ZEB1 as a function of an EMT-inducing signal (here, represented by SNAIL), as predicted by the model. We also compared these transitions as mediated by the miR-200/ZEB1/ESRP1/HAS2/CD44 circuit, with the control case, i.e., when ZEB1 self-activation is not included through these detailed pathways but instead as in earlier modeling attempts (miR-200/ZEB1 circuit).^34–36^

The response of the different circuits—miR-200/ZEB1 (hereafter referred to as the “simplified circuit”) and miR-200/ZEB1/ESRP1/HAS2/CD44 (hereafter referred to as the “extended circuit”)—to varying levels of SNAIL (mimicking an induction of EMT) is presented as bifurcation diagrams of ZEB1 messenger RNA (mRNA) levels [Figs. 1(c) and 1(d)]. Lower levels of ZEB1 mRNA (<150 molecules) denote an epithelial (E) phenotype, higher values (>600 molecules) represent a mesenchymal (M) phenotype, and intermediate values (∼200–500 molecules) denote a hybrid E/M phenotype [solid blue lines in Figs. 1(c) and 1(d)].

The bifurcation diagrams of these two circuits look remarkably similar. For low SNAIL levels, cells maintain an E phenotype. An increase in SNAIL levels drives cells toward a hybrid E/M phenotype, and a further increase induces a complete EMT, causing cells to attain an M state [black dotted upward arrows in Figs. 1(c) and 1(d)]. Moreover, for certain SNAIL levels, cells can exhibit more than one phenotype and thus can spontaneously interconvert among one another, for instance, among E and M states in the {E, M} phase [green shaded region in Figs. 1(c) and 1(d)], among E, hybrid E/M, and M states in the {E, E/M, M} phase [brown shaded region in Figs. 1(c) and 1(d)], and among hybrid E/M and M states in the {E/M, M} phase [pink shaded region in Figs. 1(c) and 1(d)]. These results suggest that ZEB1 self-activation mediated by ESRP1/CD44 and HAS2/HA/CD44 axes should be considered integral to the dynamics of epithelial-hybrid-mesenchymal transition.

### Sensitivity analysis identifies ESRP1 as a key mediator of epithelial-hybrid-mesenchymal transitions

To evaluate the robustness of the model predictions mentioned above, we performed a sensitivity analysis to parameter perturbation. Our sensitivity analysis for the simplified circuit (i.e., miR-200/ZEB1 circuit without HAS2, CD44, and ESRP1) identified ZEB1 self-activation as a crucial link. Modulating the strength of self-activation significantly affected the range of SNAIL levels for which cells could acquire a stable hybrid E/M phenotype,^36^ consistent with observations for such feedback loops enabling three states.^43,44^

**FIG. 2.**
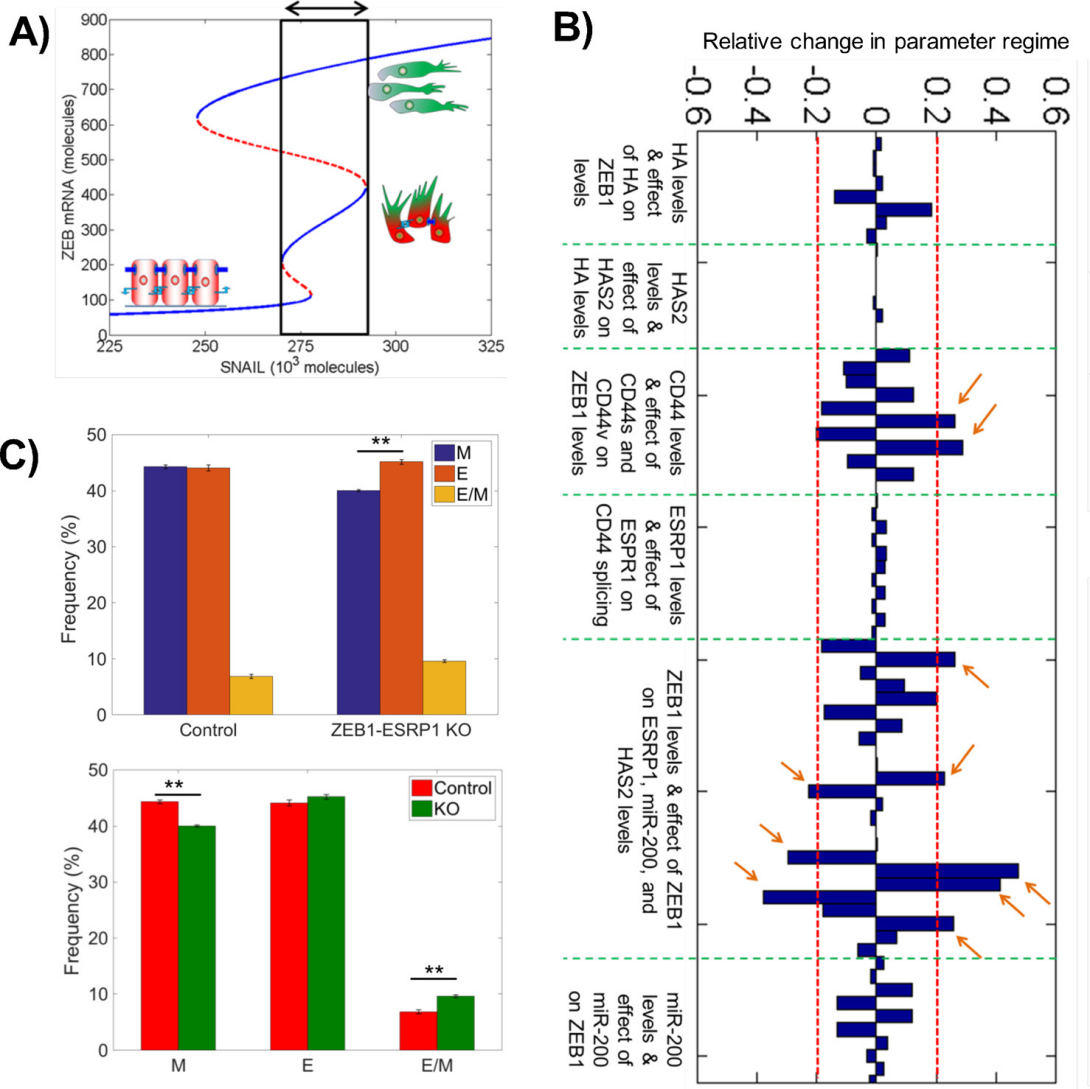
Sensitivity analysis identifies the ZEB1-ESRP1 link as a key regulator for EMT. (a) Bifurcation diagram shown in Fig. 1(d), highlighting the region for the existence of a hybrid E/M phenotype, either alone or in combination with other phenotypes. (b) Sensitivity analysis for the miR-200/ZEB1/ESRP1/HAS2/CD44 circuit [Fig. 1(b)], where the change in the width of the area highlighted in (a) is plotted for ±10% of every parameter, one at a time. Parameters are grouped based on their role in the circuit; green dotted lines denote different groups as mentioned below each group. Red dotted lines marks over ±20% change in the width of the area highlighted in (a). The model predictions are most sensitive to parameters that modulate the strength of the ZEB1-ESRP1 link (marked by arrows) (c) Solutions from an ensemble of 10 000 × 3 (n = 3) mathematical models, each with a distinct set of parameters, for two conditions—with the ZEB1 to ESRP1 node intact (control) and with ZEB1 to ESRP1 node deleted (ZEB1-ESRP1 KO). **p < 0.01 using Student's paired t-test with a two-tailed distribution. For solutions for every set of 10 000 models, the frequency of attaining E, M, or hybrid E/M state is plotted. Both plots (above and below) compare the frequency of attaining E, M, and hybrid E/M states in 10 000 control cases vs. 10 000 ZEB1-ESRP1 KO cases. They represent the same set of solutions generated from RACIPE but compared in two different ways.

To further assess which link(s) in the extended circuit (miR-200/ZEB1/ESRP1/HAS2/CD44) has (have) a significant impact on the stability of the hybrid E/M phenotype, we varied every parameter in the model for this circuit by ±10%, one at a time, and calculated its effect on the range of SNAIL levels for which cells can acquire a hybrid E/M phenotype [highlighted by a black rectangle in Fig. 2(a)]. While the model is largely robust to the parameter variation, in a few cases, we noticed a relatively larger change in the region corresponding to a stable hybrid E/M phenotype [highlighted by arrows in Fig. 2(b)]. Particularly, when the strength of inhibition of ESRP1 by ZEB1 is increased, the range of SNAIL levels for the existence of hybrid E/M decreased (Table S1). Conversely, this range increased when the inhibition of ESRP1 by ZEB1 was decreased or when the biosynthesis rates of ZEB1 were altered (Sec. S1). These results are in conceptual agreement with the sensitivity analysis for the simplified circuit.^36^ Thus, the strength of the EB1-ESRP1 interaction is likely to play a key role in controlling the stability of a hybrid E/M phenotype and the dynamics of EMT and MET.

To further characterize the role of inhibition of ESRP1 by ZEB1 in mediating EMT/MET, we applied our recently developed computational analysis method—Random Circuit Perturbation (RACIPE)^45,46^—to the miR-200/ZEB/ESRP1/CD44 circuit. For a given circuit topology, RACIPE generates an ensemble of mathematical models with randomly chosen kinetic parameters. It identifies the robust dynamical features of regulatory networks and consequently robust gene expression patterns and phenotypes that can be expected from a particular network topology. Here, we used RACIPE to generate 10 000 models for the circuit with an intact ZEB1-ESRP1 link (control case) and without this link (ZEB1-ESRP1 KO) [Figs. S1(a) and S1(b)]. The principal component analysis (PCA) of stable steady-state solutions of this ensemble of models highlights the existence of epithelial, mesenchymal, and hybrid E/M states [Fig. S2(a)]. Comparing the solutions of this ensemble of models from both these scenarios, attenuated inhibition of ESRP1 by ZEB1, i.e., effectively higher ESRP1 levels, led to a decreased propensity to attain a mesenchymal phenotype and an increased propensity to adopt a hybrid E/M state, suggesting that ESRP1 overexpression may halt or reverse EMT [Figs. 2(c), S1(a), and S1(b)]. In general, ESRP1 mediates its effects on EMT by modulating the levels of CD44s that can activate ZEB1. Thus, we also generated 10 000 models for the circuit without the link from ESRP1 to CD44s (ESRP1-CD44s KO) or without that from CD44s to ZEB1 (CD44s-ZEB1 KO). Consistently, the knockout of either link also reduced the frequency of solutions corresponding to a mesenchymal phenotype and increased that corresponding to an epithelial phenotype [Fig. S2]. Put together, the results indicate that a robust prediction of our model is that ZEB1 and ESRP1 regulate epithelial-mesenchymal plasticity in diametrically opposite ways.

### ZEB1-ESRP1 axis modulates epithelial-mesenchymal plasticity in multiple cancers

To investigate the predicted role of the ZEB1-ESRP1 axis in regulating cellular plasticity, we analyzed gene expression data from different publicly available transcriptomic datasets, using our statistical model that predicts the positioning of a given gene expression profile along the “EMT axis.”^47^ This model uses the expression of several key EMT regulators to calculate an “EMT metric” or “EMT score” on a scale of 0 (fully epithelial) to 2 (fully mesenchymal).

**FIG. 3.**
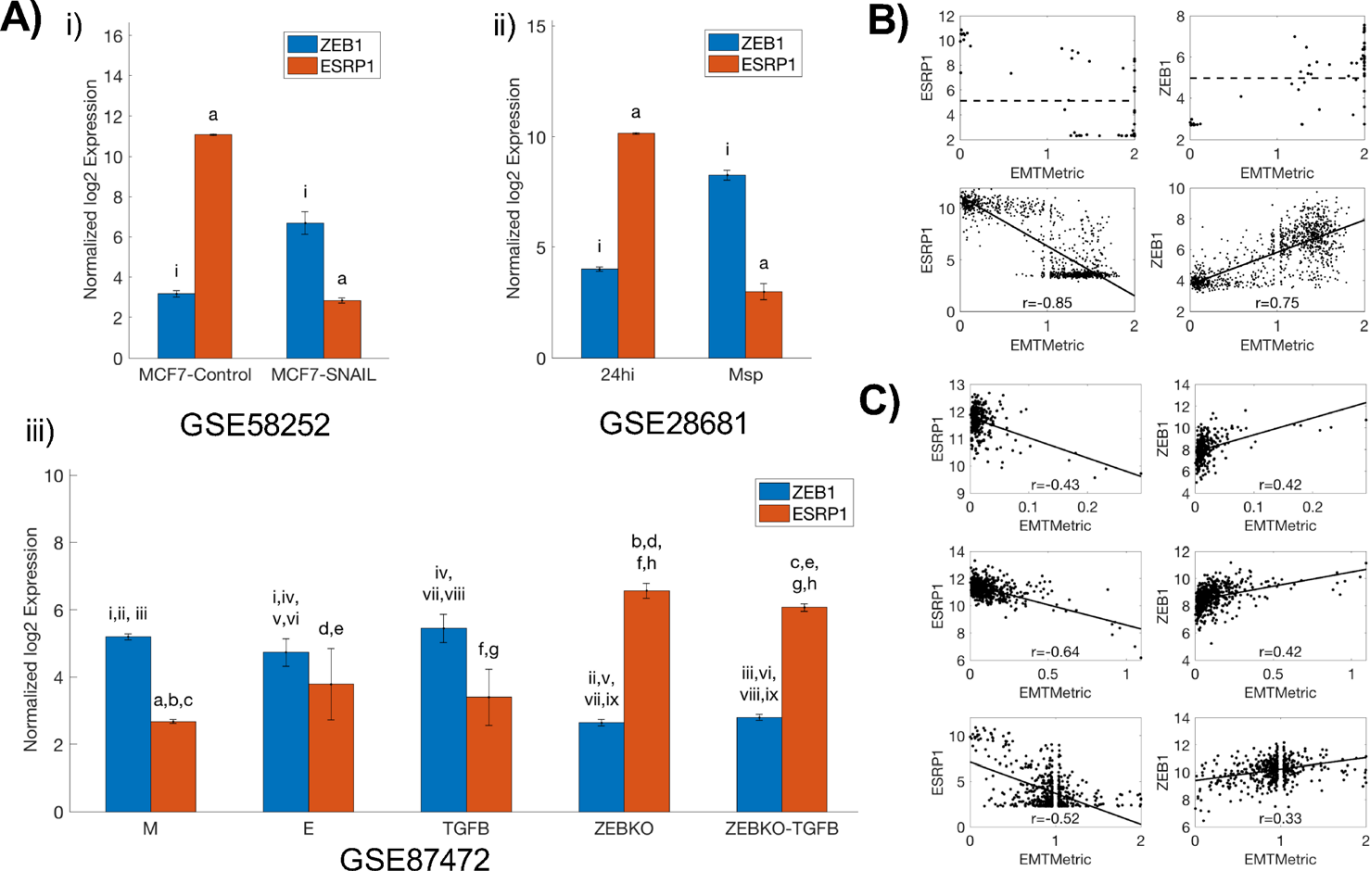
EMT metric analysis highlights the role of the ZEB1-ESRP1 axis mediating EMT. (a) Comparing the levels of ZEB1 and ESRP1 in (i) MCF7 cells with and without SNAIL transfection (n = 3 each); (ii) epithelial (24hi) (n = 2) and mesenchymal (Msp) clones established from HMLE (n = 6); and (iii) epithelial (E) (n = 8), mesenchymal (M) (n = 6), and TGFβ treated epithelial (TGFB) subpopulations from KPC mice (n = 4) and cells from KPCZ mice before (ZEBKO) (n = 14) or after TGFβ treatment (ZEBKO-TGFB) (n = 4). Lower-case roman numerals (resp. letters) indicate the pairwise statistical significance between ZEB1 (resp. ESRP1), using a one-way unbalanced ANOVA (analysis of variance). Error bars denote the standard deviation. (b) (top) Scatter plot of ESRP1 and ZEB1 vs. EMT metric in NCI-60 (n = 59). Dotted lines show the average levels of ZEB1 and ESRP1. (bottom) Correlation of ESRP1 and ZEB1 with EMT metric in the CCLE (n = 1037) cell line cohort. (c) Correlation of ESRP1 and ZEB1 with EMT metric in colon adenocarcinoma (top, n = 286), lung adenocarcinoma (middle, n = 515), and renal cell carcinoma (bottom, n = 533) TCGA datasets.

We first compared the levels of ZEB1 and ESRP1 in *in vitro* datasets. Breast cancer cells MCF7^48^ and epithelial subclones from immortalized human mammary epithelial cells HMLE^49^—that had low EMT scores^47^—expressed high levels of ESRP1 and low levels of ZEB1, whereas MCF7 cells transfected with EMT-TF SNAIL and mesenchymal subclones from HMLE exhibited a reverse trend [Fig. 3(a), i–ii; GSE58252 and GSE28681] and had higher EMT scores. This reverse trend was also observed in another study where primary tumor cells were isolated from KPC mice (a well-studied pancreatic cancer mouse model driven by Pdx1-Cre-mediated activation of mutant Kras and p53) and KPCZ mice (KPC mice with ZEB1 knockout)^50^ [Fig. 3(a), iii; GSE87472]. Tumor cells from KPC mice were highly heterogeneous, leading to establishing distinct epithelial and mesenchymal cell lines from these cells. Consistent with experimental data, epithelial cells expressed high ESRP1 and low ZEB1, while mesenchymal cells expressed high ZEB1 and low ESRP1. Treatment of epithelial cells with TGFβ (transforming growth factor beta) drove the cells towards a more mesenchymal phenotype with increased ZEB1 levels, although the observed decrease in ESRP1 levels was not statistically significant. However, cell lines derived from KPCZ (KPC mice with ZEB1-knockdown) mice were very epithelial; their EMT scores^47,50^ and thus ZEB1/ESRP1 ratio did not increase remarkably upon TGFβ treatment [Fig. 3(a), iii], consistent with the experimental observations.^50^ Similar trends were observed in pan-cancer cell line cohort NCI-60 and CCLE (Cancer Cell Line Encyclopedia).^51,52^ In the NCI-60 panel, cell lines with a higher EMT score tended to have higher ZEB1 levels and correspondingly lower ESRP1 levels [Fig. 3(b), top]. In the CCLE cell line cohort, EMT metric correlated positively with ZEB1 expression levels and negatively with ESRP1 expression levels [Fig. 3(b), bottom], suggesting that the ZEB1-ESRP1 axis may regulate EMT across cancer types. Finally, multiple TCGA datasets in lung adenocarcinoma, colon adeno-carcinoma, and renal cell carcinoma^53^—accessed from R2: Genomics Analysis and Visualization Platform (http://r2.amc.nl)—exhibited similar correlations between EMT metric and the expression levels of ZEB1 and ESRP1 [Fig. 3(c)], thus indicating the clinical relevance of the ZEB1/ESRP1 axis.

### ESRP1 knockdown destabilizes a hybrid epithelial/mesenchymal (E/M) phenotype

We have already discussed how increased ESRP1 levels can increase the range of systems that might exhibit a stable hybrid E/M state. Similarly, in our previous work, we have identified several “phenotypic stability factors” (PSFs) such as OVOL2 and GRHL2 that can stabilize a hybrid E/M phenotype. Deleting these PSFs has been shown to drive “cells that have gained partial plasticity” (i.e., in a hybrid E/M phenotype) towards a complete mesenchymal phenotype *in vitro*^25,53,54^ and *in vivo*,^55^ thus validating the predictions from mathematical modeling about their function as a PSF.^25^ Each of these PSFs forms a mutually inhibitory loop with ZEB1.^25^ From this perspective, we note that ESRP1 also forms an effective mutually inhibitory loop with ZEB1 by regulating the alternative splicing of CD44. Thus, we can make the further prediction that complete knockdown of ESRP1 may destabilize a hybrid E/M phenotype.

**FIG. 4.**
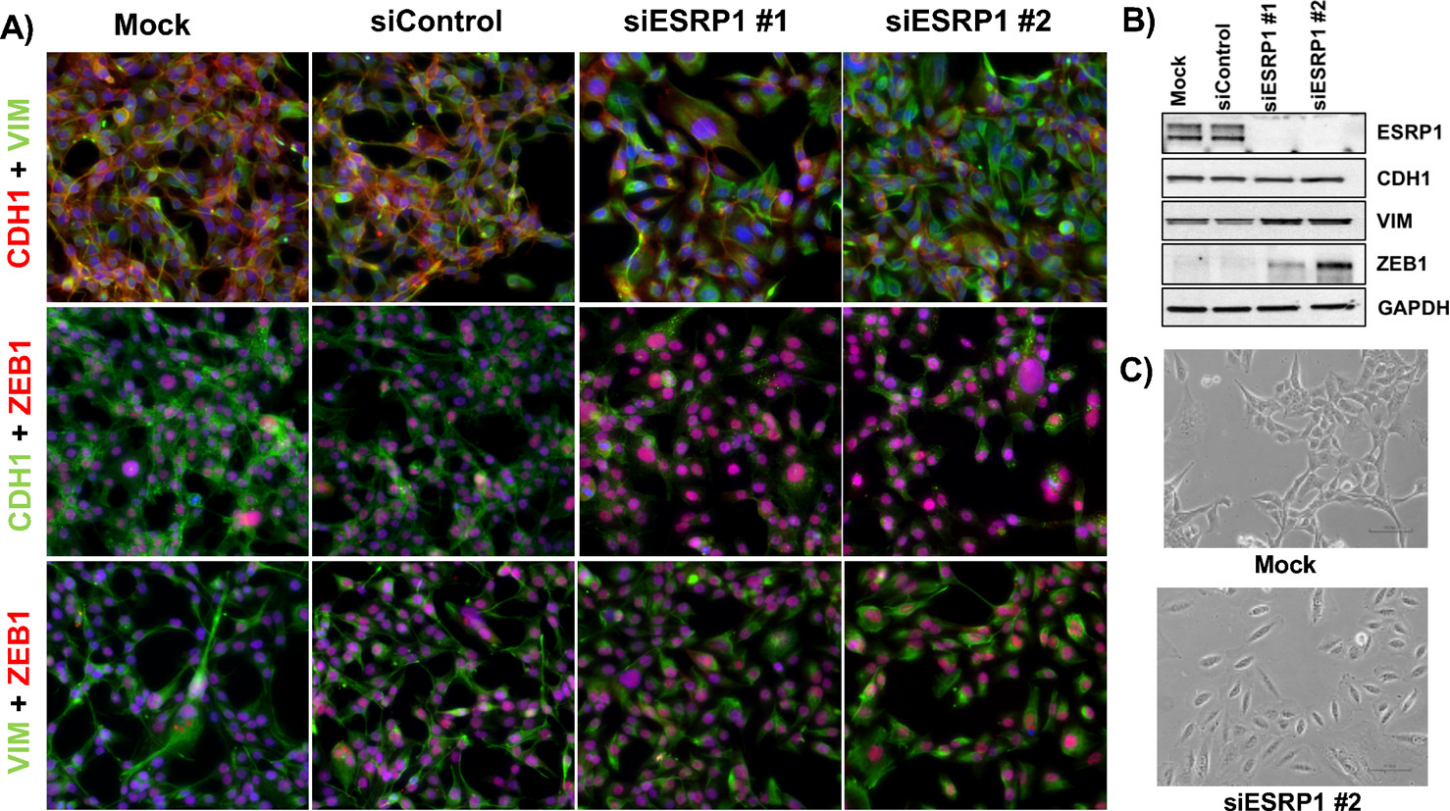
Knockdown of ESRP1 induces EMT in H1975 cells. (a) Immunofluorescence images for hybrid E/M H1975 cells—mock (leftmost column), treated with control siRNA (2nd column from the left), and treated with two independent siRNAs targeting ESRP1 (3rd and 4th columns from the left). (b) Western blot showing changes in ESRP1, CDH1, VIM, and ZEB1 levels. (c) Bright-field microscopy images of mock (top) and si-ESRP1 #2 treated H1975 cells (bottom).

To investigate this hypothesis, we knocked down ESRP1 via two independent siRNAs in H1975—lung cancer cells that display a hybrid E/M phenotype stably over multiple passages *in vitro* under normal culturing conditions.^25^ Untreated H1975 cells co-expressed E-cadherin (CDH1) and Vimentin (VIM) and showed nuclear localization of ZEB1 at a single-cell level (Fig. 4, left 2 columns). Knockdown of ESRP1 substantially increased nuclear levels of ZEB1 and disrupted the membranous localization of E-cadherin [Fig. 4(a), right 2 columns], thus indicating a transition towards a more mesenchymal phenotype. Even though the levels of E-cadherin did not change significantly [Fig. 4(b)], its membranous delocalization is considered as a hallmark of EMT.^56,57^ These observations go along with an increase in vimentin and ZEB1 on protein and RNA levels [Figs. 4(b) and S3] and an increased spindle-shape morphology [Figs. 4(c) and S3] in ESRP1 knockdown cells, further supporting a shift towards a mesenchymal state.

Next, to explore the effects of overexpressing ESRP1 in cells in a mesenchymal state, we treated MCF10A cells with TGFβ for 21 days. Previous work has shown this treatment to be necessary and sufficient to induce the various molecular and morphological changes associated with EMT.^39^ We then overexpressed ESRP1 in these cells. Overexpression of ESRP1 drove the cells towards a cobblestone-shaped morphology and a loss of ZEB1 largely (Fig. S4).

### ESRP1 expression correlates with prognosis of breast cancer patients

**FIG. 5.**
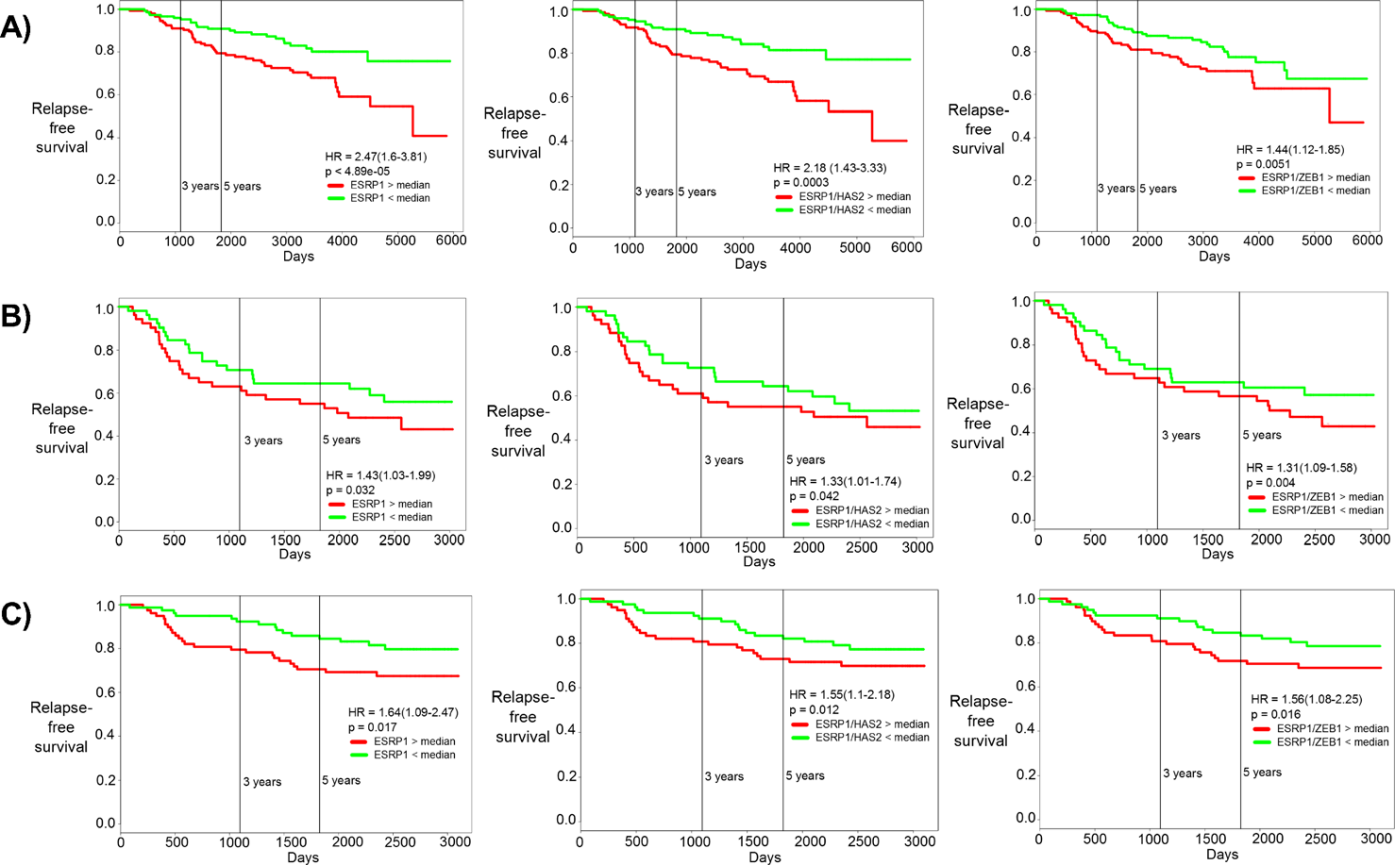
Correlation of ESRP1, ESRP1/HAS2, and ESRP1/ZEB1 with patient prognosis. Kaplan-Meier curves for relapse-free survival for (a) GSE 17705, (b) GSE 42568, and (c) GSE 1456. HR stands for the hazard ratio, i.e., the ratio of likelihood of relapse-free survival of patients with low ESRP1 as compared with those with high ESRP1. The first column is for ESRP1, the middle column is for the ESRP1/HAS2 ratio, and the right column is for the ESRP1/ZEB1 ratio. The red curve shows patients with high ESRP1 levels and green curves patients with low ESRP1 levels (separated by median).

PSFs such as GRHL2 associate with poor prognosis in breast cancer,^25,58^ motivating us to investigate the correlation of ESRP1 with patient survival rates. Higher levels of ESRP1, and a higher ratio of ESRP1/HAS2 and ESRP1/ZEB1, correlate with poor relapse-free survival (RFS) and overall survival (OS) in multiple independent breast cancer datasets [Figs. 5(a)–5(c) and S5]. In all these cases, the hazard ratio is greater than 1 (HR > 1), and the difference among the two groups is statistically significant (p < 0.05). HR > 1 implies that the patient samples with higher levels of ESRP1, ESRP1/HAS2, or ESRP1/ZEB1 tend to have relatively poor RFS or OS as compared to those with lower levels of the same. These results suggest that the ZEB1/ESRP1/HAS2/CD44 axis may play important functional roles in breast cancer progression.

## DISCUSSION

EMT, a key developmental program that plays crucial roles in many pathological conditions,^59^ is affected by multiple cross-wired cell-intrinsic and cell-extrinsic signals, including mechanical regulation through factors such as matrix stiffness.^11–13^ Hyaluronan—also known as hyaluronic acid (HA)—is a proteoglycan that forms a scaffold for the assembly of the extracellular matrix (ECM). It mediates its metastasis-promoting effects largely by interacting with various isoforms of the cell surface receptor CD44.^60^ The relative abundance of these isoforms is regulated by an alternative splicing regulatory ESRP1^39,40^ that is altered during EMT in multiple cancer types.^37,40^ HA-CD44 interactions can activate ZEB1—a key transcription factor driving EMT—which, in turn, not only inhibits ESRP1 but also upregulates HAS2 that synthesizes HA.^38,39^ ZEB1 correlates negatively with ESRP1 and positively with HAS2 in NCI-60 and CCLE cell line cohorts and in lung, breast, and pancreatic tumors,^38,39^ indicating that feedback loops operating among ZEB1, ESRP1, and HAS2 may modulate cancer cell plasticity across subtypes. Put together, this set of feedback loops and complex interactions give rise to a highly nonlinear dynamic regulation of epithelial-hybrid-mesenchymal phenotypic transition that we characterized here through an integrated theoretical-experimental approach.

Our mathematical model predicts that the nonlinear dynamics emerging from these inter-connected feedback loops can enable three phenotypes—epithelial (low ZEB1), mesenchymal (high ZEB1), and hybrid E/M (intermediate ZEB1)—that can potentially co-exist in clonal populations and interconvert spontaneously. Multiple recent studies have identified a hybrid E/M phenotype at a single-cell level in cell lines, primary tumors, circulating tumor cells (CTCs), and metastatic tumors, across cancer types.^61–63^ Cells in a hybrid E/M phenotype, in contrast to those in solely epithelial or mesenchymal states, are more likely to exhibit enhanced plasticity and tumor-initiation potential, drug resistance, and anoikis resistance *in vitro* and *in vivo.*^27–29,64–68^ Moreover, many CTC clusters—the primary harbingers of metastasis^69^—can exhibit a hybrid E/M signature.^70^ These observations drive attention toward mechanisms maintaining a hybrid E/M phenotype.^53,71–73^ Furthermore, subpopulations displaying different phenotypes (E, M, and hybrid E/M) were observed to spontaneously interconvert,^27,74^ and these transitions may even mediate interconversion among cancer stem cells (CSCs) and non-CSCs.^75^ Therefore, the different ratios of these subpopulations may vary dynamically in a given cell line. Single-cell and time-course investigation into clonal populations, using reliable live-cell reporter systems,^76^ is better suited to highlight such non-genetic heterogeneity.

Our experimental observations that ESRP1 knockdown can drive a full EMT in H1975 cells propose ESRP1 as a potential factor that can stabilize a hybrid E/M phenotype. This idea is strengthened by our computational analysis using RACIPE which shows an enrichment of the hybrid E/M phenotype when the inhibition of ESRP1 by ZEB1 is deleted (i.e., increased ESRP1 levels effectively). Finally, similar to the prognostic ability of GRHL2, OVOL, and ΔNP63α^25,58,72^—factors that can stabilize a hybrid E/M phenotype^25,36,53,55^—higher levels of ESRP1 have been associated with (a) poor OS in breast cancer, (b) poor 5-year progression-free survival (PFS) and OS in epithelial ovarian cancer samples,^77,78^ (c) poor prognosis of distant metastasis in breast cancer samples,^79^ and (d) enhanced metastasis in colorectal cancer progression.^80^ Put together, these observations support a case for ESRP1 to be referred to as the “phenotypic stability factor” (PSF). However, including ESRP1 and its interconnections with the miR-200/ZEB circuit did not change the bifurcation diagram of the circuit, as was observed for other PSFs such as GRHL2, OVOL2, and ΔNP63α.^25,36,73^

Higher levels of ESRP1, as well as higher ratios of ESRP1/ZEB1 and/or ESRP1/HAS2, can predict poor survival in multiple breast cancer datasets. This observation may appear counter-intuitive *prima facie*, given the role of ESRP1 in inhibiting EMT. However, recent studies have suggested caution against long-held universality of EMT associating with poor prognosis^27,81^ by arguing that EMT is not always a binary process. Thus, higher levels of ESRP1 observed in these datasets may correspond to a hybrid E/M phenotype, which, as per the emerging notion,^27–29,64–68^ may be more aggressive than cells at either end of the “EMT axis.” Besides breast cancer, ZEB1 has been shown to directly repress ESRP1 in non-small cell lung cancer (NSCLC),^82,83^ explaining the negative correlation between them in 22 NSCLC cell lines.^84^ Our results showing that ESRP1 knockdown alters ZEB1 levels in H1975—a hybrid E/M NSCLC cell line—suggest that this mutual inhibition between ESRP1 and ZEB1 may be functionally active in NSCLC.

Increased ESRP1 can enrich for CD44v; thus, consistent with the prognostic ability of ESRP1, higher levels of CD44v, but not high CD44s or total CD44 levels, are prognostic markers for distant metastases in lung and breast cancer.^79^ CD44v can contribute to metastasis in multiple ways, e.g., by controlling the stability of cysteine transporter xCT which enables defense against enhanced oxidative stress.^85^ Another example would be the production of an adhesive matrix—likely via anchoring to hyaluronan—to which tumor cells can attach during metastasis.^86^ Contrarily, CD44s can also accelerate metastasis by increasing ZEB1 levels through HA-dependent and/or HA-independent pathways.^38,39^ Therefore, a fine-tuned balance of CD44s and CD44v isoforms may provide additional metastatic advantages. This balance is likely to correspond to a hybrid E/M phenotype. Future studies would need sophisticated experimental models to understand spatiotemporal expression and function of CD44 isoforms during cancer progression.

In conclusion, our results demonstrate how two interconnected feedback loops of ZEB1 including ESRP1, HAS2, and CD44 govern the dynamics of EMT/MET and enable the existence of a stable hybrid E/M phenotype. ZEB1 can not only enforce its own expression through these loops but also alter its microenvironment, thus enhancing non-cell autonomous effects of EMT. Similar non-cell autonomous effects can lead to a cooperative behavior between epithelial and mesenchymal cells in establishing metastases.^87–89^ Moreover, these non-cell autonomous effects of EMT mediated by ZEB1 may help offer a plausible explanation why metastasis can be blunted largely by knockout of ZEB1^50^ but not necessarily by that of SNAIL or TWIST.^90^

## METHODS

### Cell culture

Cell line MCF10A was purchased from American Type Culture Collection (ATCC). Cells were cultured in Dulbecco's Modified Eagle's Medium (DMEM)/F12 (Invitrogen, Karlsruhe, Germany, 31331) containing 5% horse serum (Life Technologies, Darmstadt, Germany, 16050122), 20 ng/ml epidermal growth factor (EGF) (R&D Systems, Wiesbaden, Germany, 236EG200), 0.5 mg/ml hydrocortisone (Sigma, Taufkirchen, Germany, H0888#1G), 0.1 mg/ml cholera toxin (Sigma, Taufkirchen, Germany, C-8052), and 10 mg/ml insulin (Invitrogen, Karlsruhe, Germany, 12585‐014). Induction of EMT by TGFb1 (PeproTech, Hamburg, Germany, 100‐21) in MCF10A cells was performed by adding daily 5 ng/ml TGFb1 to the medium of MCF10A cultures for 21 days. The medium was replaced every second day. After 21 days, MCF10A ESRP1 cells were generated by lentiviral infection with an ESRP1 overexpression and control construct (pCDH-CMV-(ESRP1)-MCS-EF1-copGFP).

H1975 cells were cultured in RPMI 1640 medium containing 10% fetal bovine serum and 1% penicillin/streptomycin cocktail (Thermo Fisher Scientific, Waltham, MA). Cells were transfected at a final concentration of 50 nM siRNA using Lipofectamine RNAiMAX (Thermo Fisher Scientific) according to the manufacturer's instructions using the following siRNAs: siControl (Silencer Select Negative Control No. 1, Thermo Fisher Scientific), siESRP1 #1 (SASI_Hs01_00062865, Sigma-Aldrich, St. Louis, MO), and siESRP1 #2 (SASI_Hs02_00308155, Sigma-Aldrich, St. Louis, MO).

### Western blotting analysis and immunofluorescence

MCF10A cCells were rinsed once in phosphate buffer saline (PBS) and lysed in TLB. 30 *μ*g of protein was separated by SDS-PAGE (sodium dodecyl sulfate–polyacrylamide gel electrophoresis) (10%) for 1 h, 150 V and transferred to a nitrocellulose membrane by wet blotting in transfer buffer for 2 h, 300 mA at 4 °C. Membranes were immersed in antigen pretreatment solution (SuperSignal Western Blot Enhancer, Thermo Scientific) for 10 min and blocked in 5% skim milk/TBST for 30 min at room temperature. Primary antibody incubation was carried out using a primary antibody diluent (SuperSignal Western Blot Enhancer, Thermo, 46641) overnight at 4 °C. After washing with TBST (a mixture of tris-buffered saline and polysorbate 20), the membrane was incubated with the HRP-conjugated secondary antibody in 5% skim milk/TBST for 1 h at RT. Detection was carried out using a SuperSignal West Femto Maximum Sensitivity Substrate (Thermo, 34094) or an ECL Prime Western Blot Detection Reagent (Amersham, RPN2232) and a ChemiDoc imaging system (BioRad). Quantification was performed where appropriate using ImageJ and presented normalized to β-Actin levels.

H1975 cells were lysed in RIPA lysis assay buffer (Pierce) supplemented with protease and a phosphatase inhibitor. The samples were separated on a 4%–15% SDS-polyacrylamide gel (Biorad). After transfer to the PVDF (Polyvinylidene diflouride) membrane, probing was carried out with primary antibodies and subsequent secondary antibodies. Primary antibodies were purchased from the following commercial sources: anti-CDH1 (1:1000; Cell Signaling Technology), anti-vimentin (1:1000; Cell Signaling Technology), anti-ESRP1 (0.4 *μ*g/ml; Sigma), anti-ZEB1 (1:200; Abcam), and anti-GAPDH (Glyceraldehyde 3-phosphate dehydrogenase) (1:10 000; Abcam). Membranes were exposed using the ECL method (GE Healthcare) according to the manufacturer's instructions. For immunofluorescence, cells were fixed in 4% paraformaldehyde, permeabilized in 0.2% Triton X-100, and then stained with anti-CDH1 (1:100; Abcam), anti-vimentin (1:100; Cell Signaling Technology), and anti-ZEB1 (1:200; Abcam). The primary antibodies were then detected with Alexa conjugated secondary antibodies (Life technologies). Nuclei were visualized by co-staining with DAPI (4′,6-diamidino-2-phenylindole).

### Transfection of plasmid DNA

Plasmid DNA transfection was done by using the FugeneHD transfection reagent (Promega, E2311) according to the manufacturer's instructions and harvested 72 h afterwards for protein analysis.

### Antibodies

The following antibodies and dilutions were used for Western blotting: mouse anti-β-actin (Sigma, A5441; 1:5000), rabbit anti-ZEB1 (Sigma, HPA027524; 1:5000), HRP-coupled goat anti-rabbit IgG (Dianova, 111‐035-003; 1:2 50 000), goat anti-mouse IgG (Dianova, 115‐035-003; 1:25 000), and mouse anti-ESRP1 (Abnova, Heidelberg, Germany, ab140671; 1:500).

### RT-PCR (Reverse transcription polymerase chain reaction)

Total RNA was isolated following manufacturer's instructions using the RNeasy kit (Qiagen). cDNA (complementary DNA) was prepared using the iScript gDNA clear cDNA synthesis kit (Bio-Rad). A TaqMan PCR assay was performed with a 7500 Fast Real-Time PCR System using TaqMan PCR master mix, commercially available primers, FAM™-labeled probes for CDH1, VIM, ZEB1, and ESRP1, and VIC™-labeled probes for 18S, according to the manufacturer's instructions (Life Technologies). Each sample was run in triplicate. Ct values for each gene were calculated and normalized to Ct values for 18S (ΔCt). The ΔΔCt values were then calculated by normalization to the ΔCt value for control.

### Mathematical model

Section S1 contains all details of mathematical model development, analysis, and parameter estimates.

### EMT metric analysis

The EMT Metric, as previously described,^47^ was applied to various Gene Expression Omnibus (GEO) datasets. A collection of EMT-relevant predictor transcripts and a set of cross-platform normalizer transcripts were extracted for each dataset and used to probabilistically categorize samples into an element of {E, E/M, M}. To each sample *i*, there corresponds an ordered triple S_i_ = (P_E_, P_E/M_, P_M_) that characterizes the probability of group membership. Categorization was assigned based on the maximal value of this ordered triple. S_i_ was then projected onto [0,2] by the use of EMT metric. The metric places epithelial (resp. mesenchymal) samples close to 0 (resp. 2), while maximally hybrid E/M samples are assigned values close to 1.

### Ethics approval statement

No such approval is needed for this study.

## SUPPLEMENTARY MATERIAL

See supplementary material for modeling details, parameter estimation, sensitivity analysis, and other additional analysis.
